# OVERVIEW OF NIA MISSION AND RESEARCH

**DOI:** 10.1093/geroni/igad104.0972

**Published:** 2023-12-21

## Abstract

Dr. Hodes will provide an overview of NIA’s structure and mission, in addition to discussing research foci from across the Institute’s scientific divisions.

